# Neural and behavioral changes driven by observationally-induced hypoalgesia

**DOI:** 10.1038/s41598-019-56188-2

**Published:** 2019-12-24

**Authors:** Nandini Raghuraman, Yang Wang, Lieven A. Schenk, Andrew J. Furman, Christina Tricou, David A. Seminowicz, Luana Colloca

**Affiliations:** 1 Department of Pain and Translational Symptom Sciences University of Maryland School of Nursing, Baltimore, MD USA; 2 Departments of Anesthesiology and Psychiatry University of Maryland School of Medicine, Baltimore, MD USA; 3 Department of Neural Pain Sciences University of Maryland School of Dentistry, Baltimore, MD USA; 4 Center to Advance Chronic Pain Research, University of Maryland, Baltimore, MD USA; 5 Social Neuroscience Lab, Max Planck Society, Berlin, Germany

**Keywords:** Social neuroscience, Pain

## Abstract

Observing successful pain treatment in others can induce anticipatory neural processes that, in turn, relieve pain. Previous studies have suggested that social learning and observation influence placebo hypoalgesia. Here, we used electroencephalography (EEG) to determine the neurophysiological changes associated with pain relief acquired through the observation. Thirty-one participants observed a demonstrator undergo painful heat stimulations paired with a “control” cream and non-painful ones paired with a “treatment” cream, which actually were both Vanicreams. After their observation, the participants then received the same creams and stimulations. We found that the treatment cream led to lower self-reported pain intensity ratings than the control cream. Anticipatory treatment cues elicited smaller P2 in electrodes F1, Fz, FC1, and FCz than the control condition. The P2 component localization indicated a higher current density in the right middle frontal gyrus, a region associated with attentional engagement. In placebo responders, the sensorimotor cortex activity captured in electrodes C3, Cz, and C4 indicated that hypoalgesia was positively correlated with resting state peak alpha frequency (PAF). These results suggest that observationally-induced placebo hypoalgesia may be driven by anticipatory mechanisms that modulate frontal attentional processes. Furthermore, resting state PAF could serve as a predictor of observationally-induced hypoalgesia.

## Introduction

Research on placebo hypoalgesia has gained momentum in the last decade as healthcare professionals have begun to unravel how context, rather than the specific actions of a drug, can lead to beneficial outcomes^1,2^. Among the mechanisms that drive placebo hypoalgesia, expectancy plays a crucial role. Anticipation of pain relief from a treatment reduces self-reported pain intensity. Placebo hypoalgesia can be acquired in several ways, including classical conditioning, where the patient associates pain relief with a specific medication, verbal instructions, where the health provider tells the patient that a specific drug will reduce pain, and observational learning, where the patient observes pain relief in another patient while they receive the same medication (for a review, see^3^).

The ability of observation to generate treatment expectancies with an aim to alter pain perception is a fruitful area of study. Observation is a powerful way to gain information and change behaviors, accordingly. Bandura termed this process “observational learning”^4,5^. At the behavioral level, observation as well as classical conditioning and verbal suggestion have been found to influence^6–9^ or mediate^6–9^ hypoalgesia.

An advantage of electroencephalography (EEG) is the ability to record temporal dynamics of neural processing^10^. Recently, resting state peak alpha frequency (PAF) has been associated with cortical excitability rate, which influences how information is processed^11,12^ and can potentially predict pain^13^. Studies exploring painful contact-heat stimulations have identified a late event-related potential (ERP) approximately 250–350 ms after the onset of a painful stimulation in the vertex region of the brain (e.g., electrodes Cz). This component was coined as P2^14,15^. Previous studies on ERP and placebo hypoalgesia have demonstrated that placebo manipulations reduced the amplitude of pain-stimulation-induced P2^16,17^. Although anticipation of pain was thought to play a key role in shaping hypoalgesia, as far as we know, none of the previous studies examined the ERP response during the anticipation phase. A recent Magnetoencelography (MEG) study has demonstrated that, in sensors located in the frontal central brain areas, an anticipatory visual cue associated with low pain induced a smaller P2 event-related field (ERF) component compared to a higher pain anticipatory cue^18^.

Given the importance of anticipation, our study aimed to understand how and when the brain responds to observationally-induced placebo hypoalgesia. To achieve this, 31 healthy study participants (19 women) completed an observation and an experience phase while undergoing EEG acquisitions. During the observation phase, volunteers saw pictures of a demonstrator experiencing heat pain on his left forearm. The demonstrator had two colored creams (green and blue, randomized) applied that were described as treatment and control. Participants were instructed that one of the two creams had analgesic properties but were not told which one was the control and the active treatment. This distinction was acquired throughout the observational phase. During heat stimulations, the demonstrator showed a pained facial expression for the control cue and neutral facial expression for the treatment cue. During the experience phase, the same creams were applied to the participants, and they received identical levels of heat during both of the cues. Their pain ratings after each cue were recorded to determine how the anticipatory cues would modulate their pain. In line with previous result^18^, we hypothesized that observationally-induced placebo hypoalgesia would be associated during anticipatory phase with reduced frontal lobe neural activity, such as the P2 component of the evoked potentials.

## Results

### Participants characteristics

In this cohort of 31 healthy participants, we found no significant effects of sex (F_1,26_ = 0.69, p = 0.413), age (r = −0.23, p = 0.290) and race (F_1,25_ = 0.22, p = 0.804) on observationally-induced hypoalgesia (Table 1). Also, as the same demonstrator (a White male) was presented throughout the observation phase, participants completed the Implicit Association Test (IAT)^19^ to determine their racial preferences. The IAT^19^ measured differential associations of White vs. African American/Asian with good vs. bad attributes. The IAT D score was calculated as an index of each participant’s racial attitude. We tested for the possible influences of racial preference in observationally-induced placebo hypoalgesia and found no significant relationship between the IAT D score and observationally-induced hypoalgesia (r = 0.04, p = 0.829), suggesting that racial preferences did not influence observationally-induced hypoalgesia. The IRI total range was 45 to 96 with no significant relationship with observationally-induced hypoalgesia. Empathic concern subscale did not correlate with observationally-induced hypoalgesia (r = 0.22, p = 0.246 Bonferroni corrected). Neither did BES-cognitive empathy (r = −0.06, p = 0.752) nor BES-affect empathy (r = −0.27, p = 0.144), suggesting that trait empathy was not significantly associated with observationally-induced hypoalgesia when pictures of the demonstrator are shown.

**Table 1:** Participants’ characteristics.

	N/mean	%/S.D.
**Sex**		
Women	19	61.3%
Men	12	38.7%
**Race**		
White	9	29.0%
African American	8	25.8%
Asian	14	45.2%
**Age**	23.4	4.0
**Blood Pressure**		
Diastolic	74.0	8.0
Systolic	119.5	11.3
**Heart Rate**	69.7	14.0
**Height (m)**	1.70	0.10
**Weight (kg)**	72.6	16.2
**BMI (kg/m)**^**2**^	25.2	4.9

#### Observational learning

**Figure 1:**
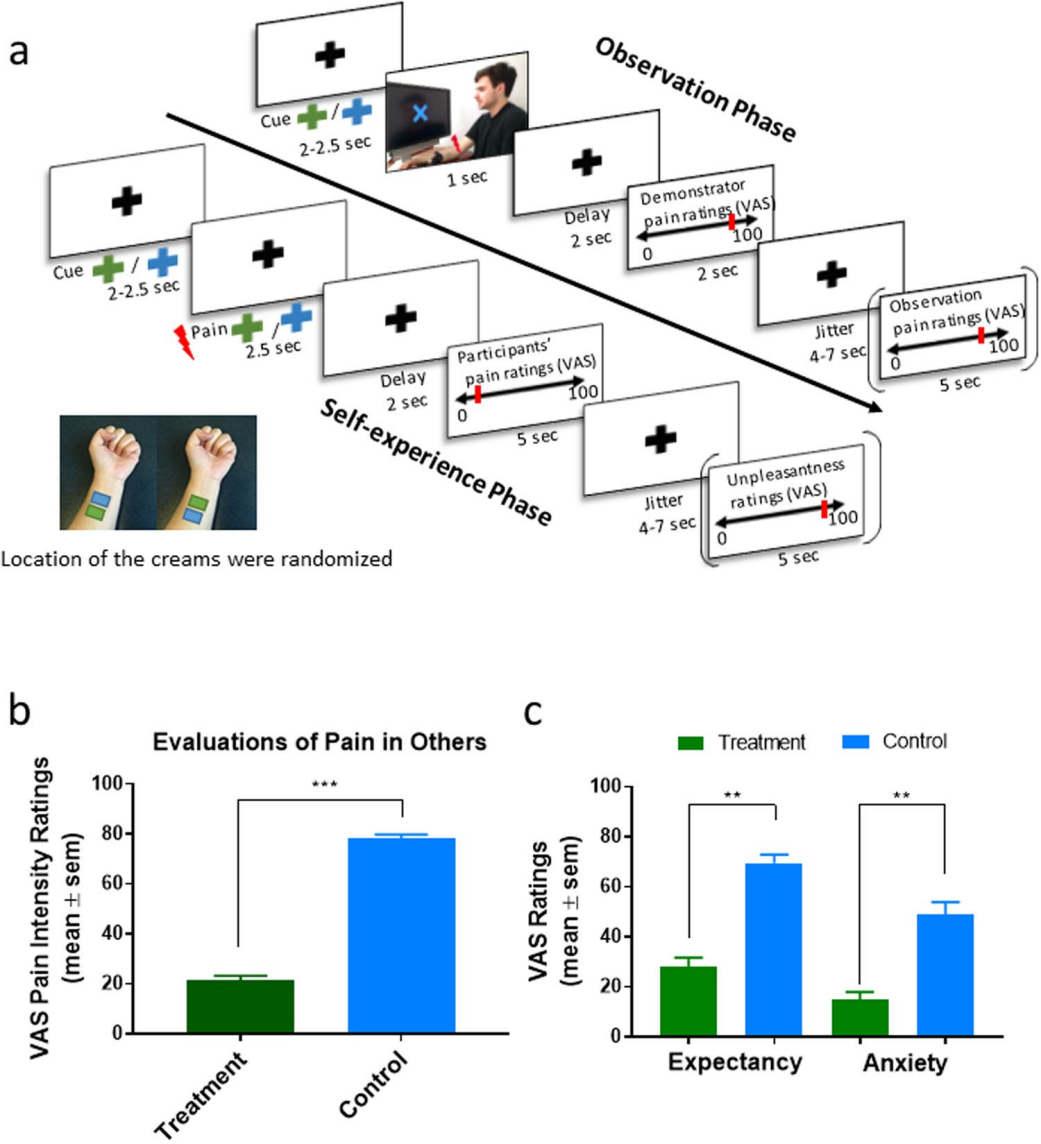
Experimental paradigm and behavioral results. (**a**) Example of a trials within the observational experience phases. (**b**) Participants rated others’ pain as lower pain intensity for treatment blocks than control blocks, suggesting that they successfully learned the association between color of the creams and the painful/analgesic experiences. (**c**) Participants felt less anxious and expected lower pain intensity for treatment blocks compared to control blocks during the experience phase. The demonstrator has granted permission to depict his image. *p < 0.05; **p < 0.01; ***p < 0.001.

During the observation phase, we asked participants to evaluate the demonstrator’s pain intensity levels at the end of 40-replicate, counter-balanced blocks of heat-pain stimuli associated with treatment and control cues. After controlling for the color (green vs. blue) and the order of cues (treatment first vs. control first), participants reported that the demonstrator’s pain intensity was significantly lower during the treatment blocks (mean = 21.35, sem = 1.84) than the control blocks (mean = 77.94, sem = 1.75, F_1,27_ = 604.78, p < 0.001, Fig. 1b). This indicated that participants successfully associated the colors of the creams (treatment vs. control) with the demonstrator’s pain experiences (lower vs. higher).

After the observation phase, participants rated their levels of anxiety and expectations of pain relief for both treatment and control conditions. The results demonstrated that the participants felt less anxious (F_1,26_ = 20.02, p < 0.001) and expected less painful experience (F_1,26_ = 42.09, p < 0.001) for the treatment block compared to control block (Fig. 1c).

#### Observationally-induced placebo hypoalgesia

**Figure 2:**
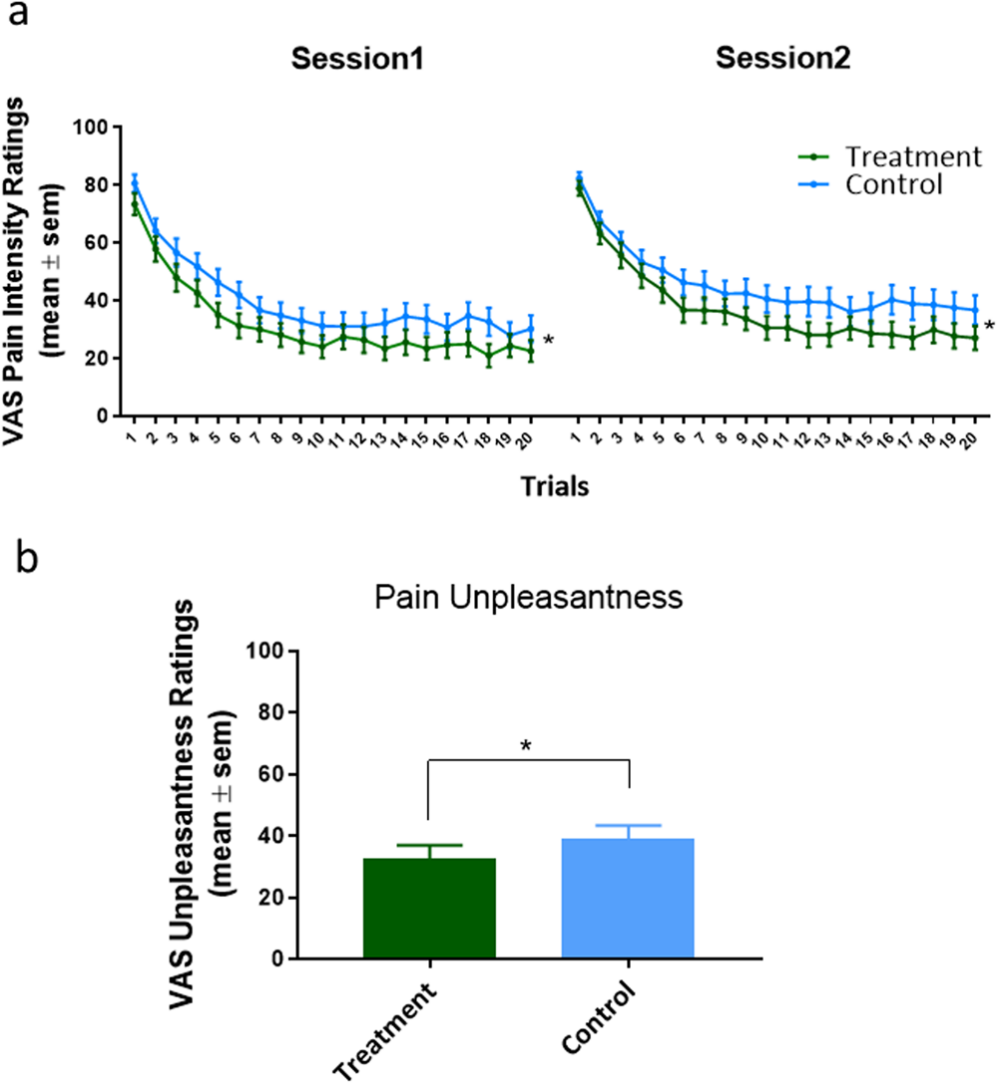
Observationally-induced placebo hypoalgesia. (**a**) There was a significant pain reduction in treatment trials as compared to control trials. The placebo hypoalgesia in session 1 was comparable to session 2. (**b**) Participants rated lower pain unpleasantness for treatment blocks than control blocks. *p < 0.05; **p < 0.01; ***p < 0.001.

A repeated-measures ANCOVA revealed a significant main effect of condition during the experience phase, where participants’ pain intensity ratings for treatment blocks (mean = 34.77, sem = 3.20) were significantly lower than control blocks (mean = 41.15, sem = 3.59; F_1,27_ = 5.18, p = 0.031, Cohen’s d = −0.361, Fig. 2a). Thus, their observation elicited significant placebo hypoalgesia. Similarly, we found that pain unpleasantness ratings were significantly lower (F_1,27_ = 5.43, p = 0.028, Fig. 2b) for treatment blocks (mean = 31.43, sem = 4.22) than ratings for control blocks (mean = 39.12, sem = 4.51, Cohen’s d = −0.405). These results were derived while controlling for cue color, block order, and thermode site (upper versus lower ventral forearm). In order to control for natural history-related changes, we used VAS ratings collected during the calibration phase when no manipulation, no verbal suggestion, and no cream application occurred. When compared to this no-intervention condition, the VAS pain reduction for treatment trials [(VAS treatment – VAS no-intervention)] was significantly more hypoalgesic than VAS pain reduction for the control trials [(VAS control – VAS no intervention)] (F_1,27_ = 5.17, p = 0.031, see Fig. S1).

In terms of pain habituation, we observed an overall non-significant trial by condition interaction (F_19,513_ = 0.522, p = 0.953) indicating that there was no extinction of observationally-induced placebo hypoalgesia from trial one to the end of 80 trials. However, we observed a significant main effect of the trials on pain intensity ratings (F_19,513_ = 72.68, p < 0.001). Post-hoc analyses applying Bonferroni correction indicated that pain intensity ratings gradually reduced across the first five trials (all p < 0.05) but did not show any differences from the 6^th^ trial to the end of each session (all p > 0.097) supporting evidence of habituation from the first five trials to the rest of the trials. A non-significant interaction of condition by session on pain intensity ratings (placebo vs. control; session 1 vs. session 2; F_1,27_ = 0.03, p = 0.876) also indicated that the observationally-induced placebo hypoalgesia between sessions 1 and 2 was comparable. There was no significant interaction between sex and condition (F_1,26_ = 0.68, p = 0.418), suggesting that men and women did not differ in observationally-induced placebo hypoalgesia. Moreover, the magnitude of this observationally-induced hypoalgesia, defined by the delta scores of pain ratings (control-minus-treatment pain ratings), were not correlated with participants’ individual pain threshold (r = 0.07, p = 0.708) or pain tolerance (r = −0.11, p = 0.567), which suggests that observationally-induced placebo hypoalgesia was independent of an individual’s pain sensitivity (see Supplementary Materials Fig. S4).

#### Resting-state peak alpha frequency

**Figure 3:**
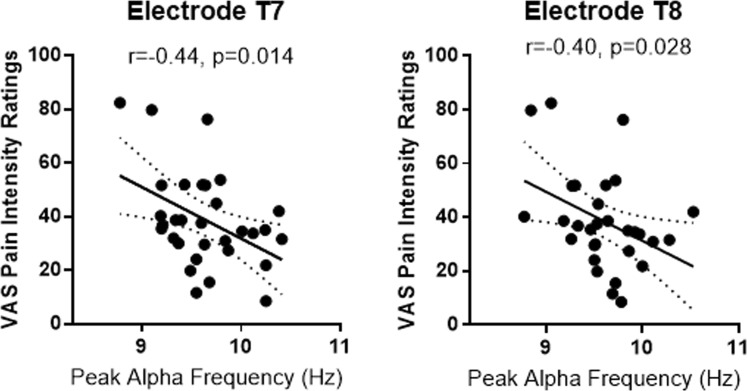
Replicating correlations between PAF and self-reported pain intensity ratings. In line with Furman *et al*.’s ^13^ findings, higher resting-state PAF was associated with lower self-reported pain intensity ratings. *p < 0.05; **p < 0.01; ***p < 0.001.

We first replicated the negative correlation between resting state peak alpha frequency (PAF) and pain intensity ratings published in the previous study^13^, confirming that higher PAF in the left and right temporal areas (T7 and T8) was associated with lower pain intensity ratings (T7: r = −0.44, p = 0.014; T8: r = −0.40, p = 0.028; Fig. 3). No significant correlations between PAF in electrodes Fp1, Fp2, C3, Cz and C4 and pain intensity ratings was observed (all p > 0.069).

In terms of observationally-induced hypoalgesia, we did not find any significant correlations between PAF and delta scores (all p > 0.386). Although PAF was negatively correlated with pain intensity ratings, it had no significant correlation with the magnitude of observationally-induced hypoalgesia.

#### ERPs elicited by anticipatory cues

Anticipation of pain relief is crucial in generating placebo hypoalgesia, therefore, we explored whether ERPs elicited by anticipatory cues could differentiate between treatment and control conditions.

**Figure 4:**
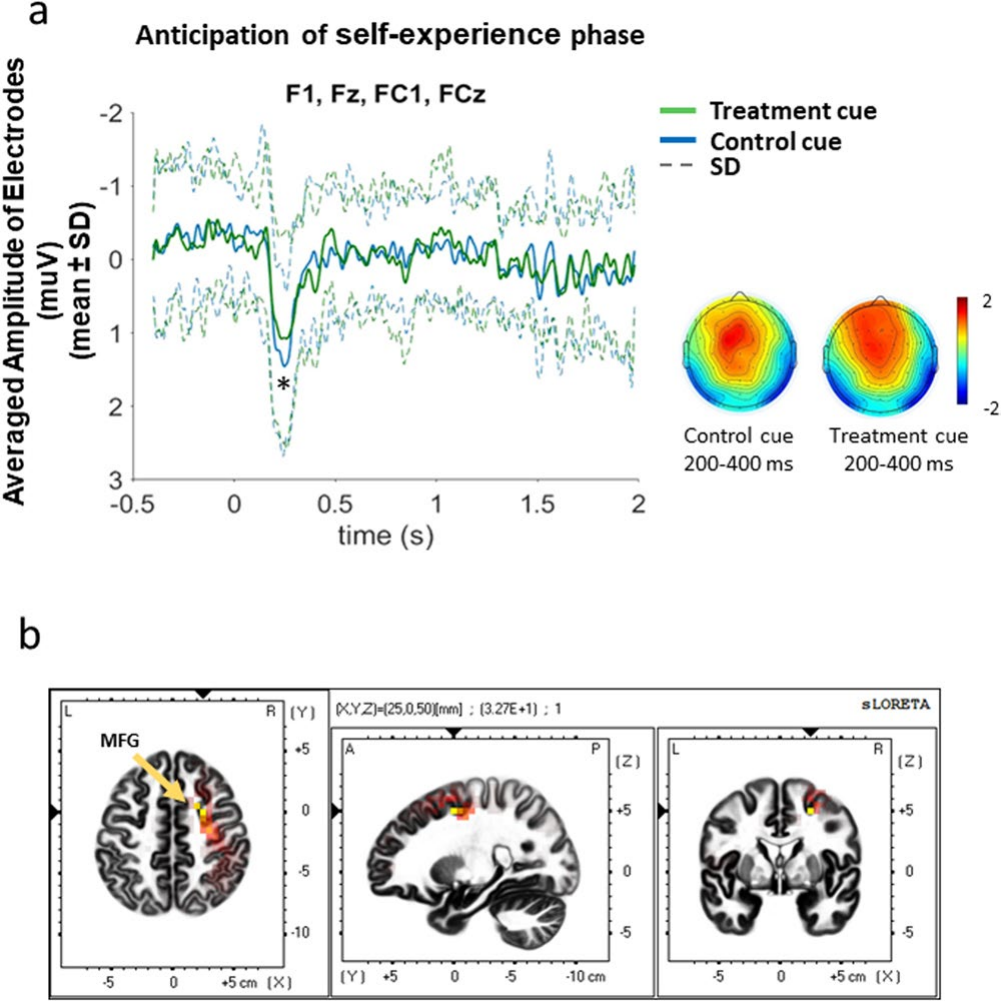
Anticipatory cues elicited P2 components in the experience phase. (**a**) Treatment anticipatory cues elicited significantly smaller P2 amplitude than control cues. The P2 component was maximal in electrodes F1, Fz, FC1, FCz at 200 ms to 400 ms after onset of the cues. (**b**) sLORETA results suggested that control cues induced marginally higher current density at right MFG (x = 25, y = 0, z = 50) than treatment cues within a time window of 200 to 400 ms after onset of the cues. *p < 0.05; **p < 0.01; ***p < 0.001.

A 2 (condition: treatment versus control) by 4 (Electrodes: F1, Fz, FC1 and FCz) repeated-measures ANCOVA controlling for cue color, cue order, and thermode site revealed a significant main effect of condition (F_1,24_ = 4.67, p = 0.041), suggesting that the anticipatory treatment cues induced a significantly smaller positive component than control cues (Cohen’s d = 0.05). This component appeared approximately 200 to 400 ms after the onset of the anticipatory visual cue and was maximal in the electrodes located in the frontal-central areas of the brain (electrodes F1, Fz, FC1, and FCz). This pattern corresponded to the time window and topography of the P2 component elicited by visual stimuli. The averaged waveforms and topography of ERPs elicited by treatment vs. control anticipatory cues are depicted in Fig. 4a. For the P2 component, no latency differences were observed between treatment (mean = 265.39, sem = 17.61) and control conditions (mean = 242.58, sem = 11.05, F_1,24_ = 0.06, p = 0.816).

More importantly, the Pearson correlations between observationally-induced hypoalgesia and the amplitude of the P2 component were negative (r = −0.41, p = 0.03). Thus, when the anticipatory treatment cues induced a smaller P2 amplitude, larger placebo hypoalgesia was observed. On the contrary, no significant correlations were observed between the P2 amplitude elicited by the anticipatory control cues and observationally-induced hypoalgesia (r = −0.29, p = 0.130). P2 amplitude changes during the anticipatory phase did not correlate with self-reported expectations (placebo trials, r = −0.01, p = 0.965 and control trials, r = 0.03; p = 0.895) that were rated before the test phase.

#### Source localization for the P2 component

Since the P2 components for treatment and control conditions differed in their amplitudes within 200 to 400 ms after onset of the cues, we limited our source analyses within this time window and calculated the image of control-minus-treatment with voxel-by-voxel t values. There was a marginally significant greater activation of the right middle frontal gyrus (coordinates: X = 25, Y = 0, Z = 50) in response to control anticipatory cues as compared to treatment cues (p = 0.087, Fig. 4b).

#### Alpha band suppression associated with painful stimuli

The EEG recordings associated with the painful stimulations were decomposed into time frequency representations. A non-significant main effect of the condition (F_1,27_ = 1.25, p = 0.274) indicated that there was no difference in alpha band mean power between treatment and control blocks (see Supplementary Materials Fig. S5). Furthermore, there was no significant correlation between the scores of control-minus-treatment alpha band mean power and observationally-induced placebo hypoalgesia (r = −0.14, p = 0.460).

#### Neural profiles of placebo responsiveness

**Figure 5:**
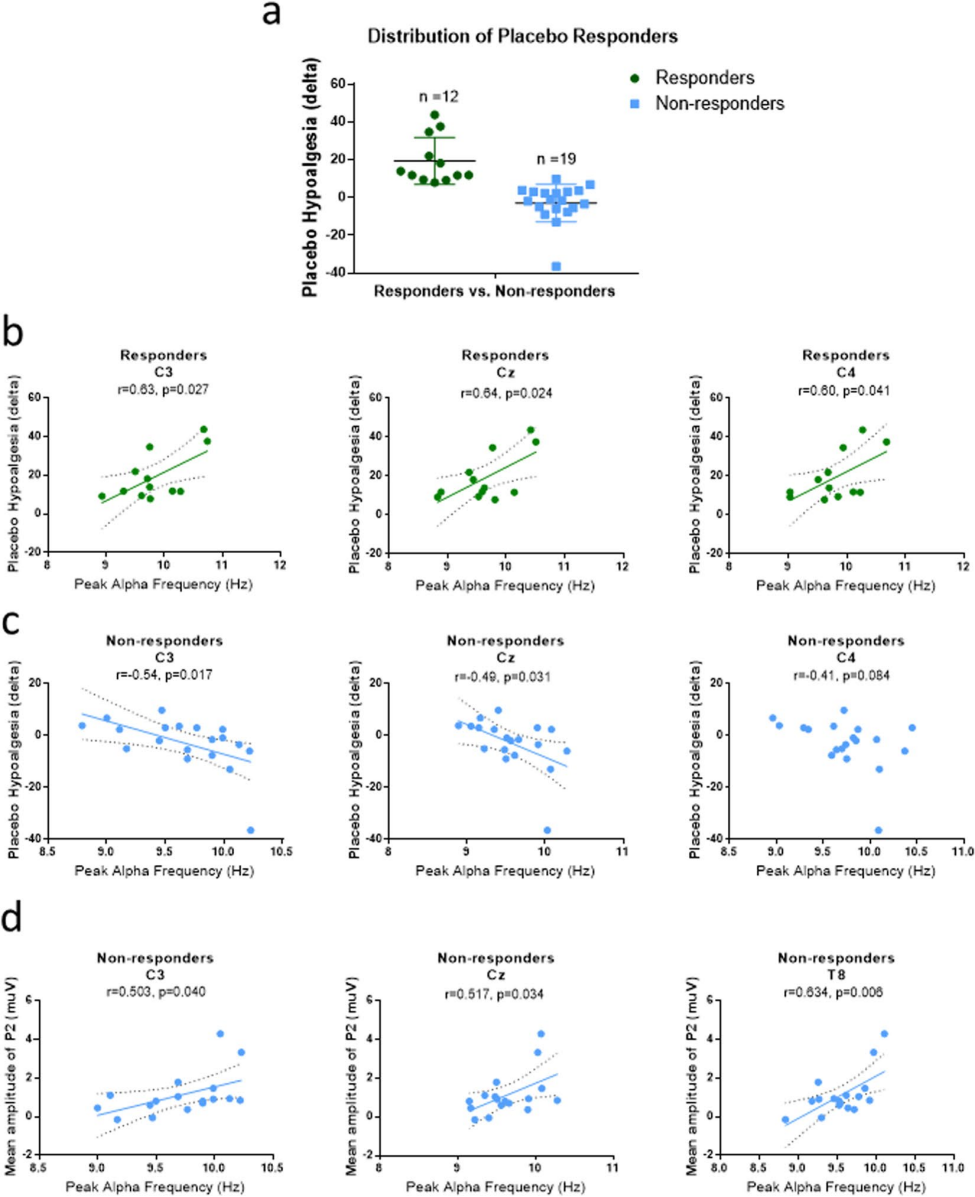
Placebo responders and non-responders differences in PAF associations with placebo hypoalgesic responses. (**a**) 12 out of 31 participants were identified as placebo responders. (**b**) In the placebo responder cohort, faster PAF was positively associated with placebo hypoalgesic scores. (**c**) In the non-responder cohort, slower PAF was negatively associated with placebo hypoalgesic scores. The correlations remained significant by removing the outliers (C3: r = −0.508, p = 0.031; Cz: r = −0.472, p = 0.048). (**d**) Within non-responders, slower PAF was associated with larger P2 amplitudes elicited by anticipatory treatment cues.

Not everyone responds to placebo manipulations. In the current cohort, 12 out of 31 participants were determined as placebo responders (responding rate 38.7%, Fig. 5a) based on permutation tests comparing their pain intensity ratings during treatment and control blocks (cut-off set as p = 0.05). As expected, placebo responders displayed significantly greater observationally-induced hypoalgesia (mean = 19.36, sem = 3.59) than non-responders (mean = −2.93, sem = 2.29, t_29_ = 5.51, p < 0.001). Placebo responders and non-responders did not differ in sex (χ^2^ = 1.55, p = 0.213) or race (χ^2^ = 0.18, p = 0.913). Moreover, placebo responders did not differ from non-responders in trait empathy, as measured by IRI scores (t_29_ = 0.44, p = 0.662).

In order to examine the potential neurophysiological factors that could distinguish observationally-induced placebo responders from non-responders, we explored the correlations between resting-state PAF in electrodes Fp1, Fp2, C3, Cz, C4, T7, T8^20^ and observationally-induced hypoalgesia. The results indicated a clear and distinct pattern between placebo responders and non-responders. Specifically, within placebo responders, higher PAF at C3 (r = 0.63, p = 0.027), Cz (r = 0.64, p = 0.024) and C4 (r = 0.60, p = 0.041, Fig. 5b) were associated with larger observationally-induced hypoalgesia. However, within non-responders, higher PAF at C3 (r = −0.54, p = 0.017) and Cz (r = −0.49, p = 0.031) were associated with smaller observationally-induced hypoalgesia. Within non-responders, the PAF at C4 showed a trend of negative correlation with observationally-induced hypoalgesia (r = −0.41, p = 0.084, Fig. 5c).

Furthermore, we explored the association between resting state PAF and treatment-cue-elicited P2 amplitude within responders and non-responders, respectively. Within responders, we did not observe any significant correlations between resting state PAF and the P2 component (all p > 0.442). However, within non-responders, we found that higher PAF at C3 (r = 0.503, p = 0.040), Cz (r = 0.517, p = 0.034), and T8 (r = 0.634, p = 0.006) were associated with greater P2 amplitudes, suggesting that non-responders have a higher information processing rate and more attentional engagement in response to anticipatory cues (Fig. 5d).

## Discussion

In this study, we sought to explore the neurophysiological correlates of observationally-induced hypoalgesia. Behaviorally, we observed that participants reported significantly lower pain ratings in response to a treatment cue compared to a control cue. This behavioral difference was induced by observing other people experiencing pain relief under similar experimental conditions. In terms of ERP results, we found that treatment anticipatory cues elicited smaller P2 components than control cues. More importantly, smaller P2 amplitudes elicited by treatment anticipatory cues were associated with larger observationally-induced hypoalgesia. Placebo responders and non-responders displayed distinct patterns in terms of the associations between observationally-induced hypoalgesia and resting state PAF. No EEG changes were detected in the test-phase despite the behavioral significant hypoalgesia suggesting that the heat model and the EEG techniques we used may not be specific and sensitive enough to detect these changes.

Colloca and Benedetti^21^ were the first to report a modulation of placebo hypoalgesia induced by observing a therapeutic treatment in a demonstrator. In line with this pioneering study^21^, we replicated the evidence that individuals reported pain reductions simply after observing beneficial treatment outcomes in others. Observationally-induced hypoalgesia was independent of the color of the creams (green versus blue), the order of the anticipatory cues, or the site of thermode (upper versus lower left ventral forearm). Additionally, we found the magnitude of pain reduction remained comparable in sessions 1 and 2, meaning that the observationally-induced hypoalgesia did not decrease, even at the end of 80 pain stimulations. These results align with previous research on video observation and placebo hypoalgesia^22^. Moreover, individual differences in pain threshold and pain tolerance did not influence the magnitude of observationally-induced hypoalgesia.

At the level of neurophysiological measurements, during the test phase, the anticipatory treatment cue elicited a significantly smaller P2 component than control cue at approximately 200 ms to 400 ms after the cue onset, and this P2 was maximal in the electrodes located in the frontal-central area of the brain. The visual-stimuli-induced P2, which has been found to be larger for threatening images compared to neutral ones^23,24^, is considered to reflect early attentional processing^25^. This implies that the participants showed reduced attentional engagement in the early processing of anticipatory treatment cues than control cues. Similar to our findings, a recent MEG study showed that an anticipatory cue for lower pain elicited smaller event-related field (ERF) P2 component than higher pain anticipatory cues^18^.

We further explored the potential sources of the observed P2 component using sLORETA. The source localization results indicated the control anticipatory cues tended to show a higher current density than the treatment cues in right middle frontal gyrus (MFG, coordinates: X = 25, Y = 0, Z = 50), a region which has already been found as one of the sources of P2 component in a simultaneous EEG-fMRI study^26^. Activation of right MFG regions has been associated with attentional processing in cognitive tasks^27–29^ and is connected to attentional networks during resting state^30,31^. This evidence highlights our results, in which attentional engagement was reduced at early stages of processing treatment cues, compared to control cues. More importantly, we found that the smaller P2 amplitudes elicited by treatment cues were associated with larger observationally-induced placebo hypoalgesia. Thus, the reduced attentional engagement for treatment cues was associated with greater observationally-induced placebo hypoalgesia.

Similar to a previous study that employed a video to induce placebo hypoalgesia^22^, we did not observe a significant correlation between empathetic concern and magnitude of observationally-induced hypoalgesia. This was likely due to using pictures of the demonstrator, rather than live face-to-face observation, to induce placebo hypoalgesia.

It should be noted that not every participant responded to placebo manipulations with a reduction of pain. In line with a recent study^32^, we performed a permutation test that offered the advantage of accounting for trial-by-trial variability across pain ratings to identify placebo responders. The responding rate in our current study was 38.7%, which is comparable to what has been reported in a review 34% placebo responding rate^33^. There was a distinct pattern between responders and non-responders regarding the link between resting state PAF and observationally-induced hypoalgesia. The resting state PAF, which has the maximal power within 7.4–12 Hz, has been considered to reflect shifts in the cortical excitability and information-processing rate^13,34,35^. Recently, the resting state PAF has also been linked to subjective pain reports^13,20^. The current results indicated that higher pain-free state PAF was associated with lower pain intensity ratings, which was in line with Furman *et al*.’s findings^13^. We found that higher resting state PAF, suggestive of brain faster information processing^13,34,35^, was positively associated with greater placebo hypoalgesic scores in the placebo *responders*. Conversely, faster brain information processing in the *non-responders* was negatively associated with observationally-induced hypoalgesia. Moreover, non-responders presented with higher PAF in association with the anticipatory-cue-elicited P2 amplitude, suggesting that the faster information processing of the non-responders may indicate high attentional engagement that lead to lesser observationally-induced hypoalgesia. The distinct patterns between placebo responders and non-responders of the PAF-placebo hypoalgesia associations suggest that not only does the information processing speed matter in formation of observationally-induced hypoalgesia, but also the core nature of the information, per se. Together, our findings suggest that placebo response status should be considered when examining the association between information processing rates and observationally-induced hypoalgesia.

Moreover, we observed an Alpha suppression at the level of the occipital areas of the brain (P1 and P3) in both control and treatment conditions at latencies of about 500 to 1300 ms (Fig. S5), suggesting cortical activation in response to pain. Previous studies have found that pain-induced alpha band activities were closely related to the intensity of painful stimuli^36–38^ and the suppression of alpha band activities, known as a reflection of cortical excitability, has been observed in the sensorimotor and occipital cortex at latencies of about 300 to 1000 ms in response to experimental pain (see review^38^). In the current study, likely due to using the same intensity pain stimulation for both treatment and control conditions, we failed to observe differences in the alpha band mean power. Alpha band suppression might not be as sensitive to placebo manipulations as previously thought^17^.

The current study has some limitations. First, this study employed a relatively long, 2-second painful stimulation. Specific durations of laser^39^ and contact heat^40^ stimulations have been associated with reduced ERP responses (e.g. shorter painful stimulation in the range of milliseconds larger ERP responses). Therefore, the 2-second contact heat painful stimulations might limit the possibility of observing a pain-related ERP during the test phase of this study. Future studies should use shorter duration of the painful stimuli (e.g., pulse mode) to explore whether pain-related potentials (e.g., N2 and P2 component) are different between treatment and control conditions. Secondly, the sLORETA algorithm uses a relatively low spatial resolution^41,42^ to identify the source of the P2 component. Alternative algorithms or methods, such as fMRI, should be employed to increase the spatial resolution of the data. Thirdly, we cannot exclude sex, race, and age differences because the study is not fully powered to address the influence of demographic features on observationally-induced analgesia and larger studies are needed to explore psychosocial factors influences. Fourth, because we used an EEG approach, we were limited to use very simple observational scenes (e.g. pictures of the demonstrator) that were repeated numerous times. To further understand the potential clinical relevance of observationally-induced placebo hypoalgesia, studies should use more ecological and realistic clinical contexts. Finally, we adopted a within-subjects design with control and treatment trials counterbalanced across study participants for color, area of cream administration, pre-test participants’ expectation ratings, and neutral instructions related to the active and control creams. Computerized Visual Analogue Scales were used to minimize reporting biases. Future studies may include a trial-by-trial measurement of expectations for the test phase to better understand and model computational approaches for the role of expectations in observationally-induced hypoalgesia.

In conclusion, this study demonstrated that merely observing other people experiencing pain relief can induce significant hypoalgesia under similar conditions via modulation of anticipations. Most importantly, anticipatory treatment cues elicited smaller P2 components than control cues, and these smaller P2 amplitudes were associated with larger placebo hypoalgesia. Thus, it is likely that anticipations and attentional engagement during the anticipation of an upcoming event played a critical role in inducing observationally-induced hypoalgesia. EEG measurement of both anticipatory P2 and resting PAF might provide predictive information about the formation of subsequent observationally-induced placebo hypoalgesia and, as such, may serve as markers of placebo responsiveness upon replication of these information. Overall, mechanistic knowledge about the anticipatory neural processes associated with observationally-induced pain relief might lead to novel developments in therapeutic strategies for pain, anxiety and other outcome.

## Methods

### Participants

This study was designed to explore the neural processes of observationally-induced hypoalgesia using EEG and thermal heat stimuli. Forty-three participants were recruited for this study, of which data from 31 participants (23.4 ± 4 years age range, 19 females) were deemed complete and analyzable. Twelve were excluded due to misinterpretation of instructions and inability to complete the EEG montage due to logistical issues (e.g., thickness of hair). Participants were phone-screened as well as screened in person to confirm their eligibility as a healthy volunteer. Specifically, participants were excluded based on the following criteria: left-handedness; impaired hearing; color blindness; any allergies or sensitivities to creams and/or food colorings; any history of chronic pain; current ongoing pain; neurological, cardiovascular, pulmonary, kidney and liver diseases; psychiatric disorders; and/or use of pain and other medications. A drug test detecting the use of amphetamines, buprenorphine, oxazepam, cocaine, methylenedioxymethamphetamine, methamphetamine, morphine, opioids, oxycodone, phencyclidine, propoxyphene, notriptyline and cannabinoids was completed before starting the experimental procedures. Participants with positive tests were excluded from the study. All participants were negative for the drug tests. The University of Maryland, Baltimore Institutional Review Board (IRB) approved the study (Prot. HP-#00069094) and all participants gave written informed consent. All procedures were conducted in accordance with the Declaration of Helsinki and with the relevant guidelines and regulations. A compensation of 100 dollars was given at the completion of the study. Since deceptive information was used during the procedure, participants were debriefed at the end of their experimental session using a study exit form that detailed the full nature of the study and the involvement of deception (see Suppl. Materials). Participants were offered the chance to withdraw their data from the study, but none did.

### Heat pain stimulation

Painful thermal heat stimuli were applied and delivered using a CHEPS thermode (PATHWAY System, Medoc, Ramat Yishai, Israel). The participants performed a pain sensitivity assessment using the limits paradigm^43^ followed by a pain calibration phase. During the pain calibration, they rated pain intensity for a series of 8 painful heat stimulations which lasted for 6 seconds each. Participants reported their pain intensity using a VAS ranging from 0 = no pain to 100 = maximum tolerable pain. This allowed a tailored, moderate level of pain (i.e., VAS ratings of 50–60) to be used for the experiment. The mean temperature used for the experience phase was 46.19 °C (sem = 0.20 °C, ranging from 43 °C to 48.5 °C). The temperature ramped up from 32 °C (baseline) with an increasing rate of 70 °C/s, maintained plateau for 2 seconds, and ramped down to baseline with a decreasing rate of 40 °C/s. The mean ramp-up time was 202.77 ms (sem = 2.84), and the mean ramp-down time was 354.84 ms (sem = 4.98).

### EEG recording

The EEG data were collected using a 64-channel Brain Vision actiCAP system (Brain Products GmbH, Munich, Germany) labeled in accordance with an extended international 10–20 system. All electrodes were referenced online to FCz site. EEGs were amplified with a DC~100 Hz band-pass and continuously sampled at 500 Hz. The 64-Channel electrode cap (Brain Vision actiCAP system, Brain Products GmbH, Munich, Germany) was set up and placed on the participant’s head. A high-viscosity gel was applied and electrode impedances were maintained below 5 kΩ.

### Experimental procedures

The experiment took place at the University of Maryland School of Nursing Clinical Suites during a single session. The participants were given an overview of the study procedures after completing the drug test. They were informed that we wanted to investigate differences in the neural processes driving observation of someone else’s pain as compared to their own pain experience. Vitals (blood pressure, heart rate, height, and weight) were collected before starting the study procedures to assist with study monitoring (Suppl. Table S1).

The participants were then instructed about the experimental tasks. They were informed that the experiment would be divided into two phases – the observation phase and experience phase. A treatment comprised of two creams was applied to their left forearm. Both creams, in reality, were VanicreamTM, a hypoallergenic cream that is free of dyes, fragrances, masking fragrances, lanolin, parabens, and formaldehyde and which is routinely used as a vehicle by pharmaceutical companies. This cream was colored in either green or blue using FDA-approved food dyes. To establish a treatment context, the participants were instructed that one of the creams had analgesic properties, but were not told which one was the control and the analgesic treatment. They were also not told how efficacious the analgesic cream would be. This allowed the participants to learn about the supposed effectiveness of the creams by observing the analgesic experience of the demonstrator in the visual cues. The color of cream was counterbalanced to further avoid that participants could link the efficacious cream to the color prior to the observational phase. In fact, the color that was associated with treatment and the location of the application (upper vs lower ventral forearm, as shown in Fig. 1a) was randomized across participants.

### Observation phase

This study had a within-subjects design, with each participant going through both the placebo and control manipulations. Before the observational phase, a resting state EEG was acquired for six minutes total - with three minutes each of closed and open eyes. Then, all of the participants started with the observation phase, which consisted of two blocks of 20 trials each of the treatment and control cues. The blocks were randomized across participants (see Fig. 1a). During each trial, participants started by seeing a colored cue (green or blue, between 2–2.5 s) indicating the color of the cream on which a demonstrator would receive the heat stimulus. Then, they observed a picture of the demonstrator (1 s). Here, the demonstrator showed a painful facial expression during the control block and a neutral facial expression during the treatment block. After a delay (white cross, 2 s), the pain rating of the demonstrator was showed on a Visual Analogue Scale (VAS) from 0 = no pain to 100 = maximum tolerable pain (2 s). For the treatment condition, the pain rating was randomly presented on the VAS between 10–30 and between 70–90 for the control condition. This was followed by an inter-trial interval (4–7 s). At the end of each observation block, the participants were asked to rate the observed pain of the demonstrator on a VAS anchored from zero to 100 (5 s).

### Experience phase

After the observation phase, participants answered four questions regarding their expectations of pain and anxiety for both conditions. Similar to the observation phase; the experience phase contained two sessions with each including two blocks of 20 trials each of the treatment and control cues. During each trial, an anticipatory cue (green or blue) indicating the upcoming heat stimuli (between 2–2.5 s), followed by the stimulation itself (2 s, with an additional 0.5 s due to the time required for the thermode to ramp up to temperature). After a delay (2 s), participants rated their pain intensity on a VAS from 0 = no pain to 100 = maximum tolerable pain (5 s). The trials ended with an inter-trial interval (4–7 s). At the end of each block, the participants rated their pain unpleasantness on a VAS from 0 = no unpleasantness to 100 = maximum tolerable unpleasantness (5 s). The heat pain stimuli were kept constant for both the conditions and was derived from a previously-performed pain calibration corresponding to a moderate pain level (i.e., VAS ratings of 50–60).

The pain intensity rating (VAS) data was acquired using Eprime v2 (Psychology Software Tools, Sharpsburg, PA, USA). Participants operated a Celeritas Fiber Optic Response System in order to provide responses (Psychology Software Tools, Sharpsburg, USA).

### Implicit assessment testing

At the end of the experimental session, participants completed the Implicit Association Test (IAT)^19^ to determine their racial preferences. As noted in previous studies^44,45^, participants may experience different pain levels based on their preference for within-group individuals (e.g., people from the same race) and out-of-group individuals (e.g., people from another race). In order to rule out this confound, we applied the IAT to measure participants’ racial attitudes toward White vs. African American/Asian races. The D measure was calculated as an index of each participant’s racial attitude. Positive D values indicated faster sorting of African American/Asian with bad and European American with good, suggesting an implicit preference for individuals of European American race. Negative D values indicated faster sorting of African American/Asian with good and European American with bad, suggesting an implicit preference for individuals of African American/Asian race. A value of 0 indicated no relative preference.

### Empathy measurements

In order to control for the individual differences, we measured participants’ empathy using the Interpersonal Reactivity Index (IRI)^46^ and The Basic Empathy Scale (BES)^47^. The IRI assessed reactions of one person to the observed experience of another. It contains 4 subscale including Perspective Taking (the tendency to spontaneously adopt the psychological point of view of others), Fantasy (taps respondent’s tendencies to transpose themselves imaginatively into the feeling and action of fictitious characters in books, movies, and plays), Empathic Concern (assesses “other-oriented” feelings of sympathy and concern for unfortunate others), and Personal Distress (measures “self-oriented” feelings of personal anxiety and unease in tense interpersonal settings). The IRI total score was used in the related analysis. The BES assessed individual’s empathy with 2 subscales: cognitive empathy and emotional empathy subscale.

### Statistical analysis

#### Behavioral data analysis

To determine if significant placebo hypoalgesia had been developed through observation, similar to previously studies^21,22,48^, we analyzed behavioral results primarily consisting of pain intensity ratings recorded during the experience phase. We used a repeated measures ANCOVA to analyze the differences between the ratings in the treatment and control conditions with condition (treatment vs. control) and trial as within-subjects factors. The color of treatment cue (green vs. blue), order of blocks (treatment first vs. control first), and cream site (treatment cream on top vs. control cream on top) were set as covariates. We also investigated the association between empathy and observationally-induced hypoalgesia (delta scores of the mean of pain intensity ratings for treatment trials subtracted from mean of pain intensity rantings for control trials) using Pearson’s correlation test (2-tailed significance). Cohen’s d^49^ was calculated to determine the effect size of observationally-induced hypoalgesia.

All significances were set at p = 0.05 and the analyses were conducted using IBM® SPSS Statistics software version 22.

#### Resting state peak alpha frequency (eyes closed)

The EEG recordings acquired before the experimental task with three minutes of closed eyes were preprocessed using the EEGLAB toolbox^50^. The first step involved band-pass filtering the EEG with a cut-off of 0.5 Hz to remove the drift of baseline. The Surface Laplacian (SL) was used to transform the scalp-recorded EEG into estimates of radial current flow. The direction of the radial currents was denoted with a positive and negative sign. The positive values represent the flow of current from the brain to the scalp (sources) while negative values represent the flow from the scalp to the brain (sinks)^51^. After rebuilding FCz electrode, independent component analysis (ICA) was done to correct for vertical and horizontal eye movements as well as muscle artifacts.

Time frequency representation were generated using the FieldTrip toolbox^52^ in Matlab. The EEG recorded during the three minutes (hundred and eighty seconds) eyes closed was segmented into five seconds epochs and power spectral density in the 2–40 Hz range was derived for each epoch in 0.2 Hz bins. The center of gravity (CoG) method was used to derive the peak alpha frequency where CoG is calculated using the following formula: $CoG={\sum }_{i}^{n}fi\ast ai/\,{\sum }_{i}^{n}ai$, fi is i-th frequency bin including and above 7.4 Hz, n is the number of frequency bins between 7.4 and 12 Hz^20^, and ai is the spectral amplitude for fi. The averaged PAF across epochs was then identified as the peak frequency^13^. To explore the associations between PAF during resting state and the observationally-induced placebo hypoalgesia in the current study, we performed Pearson correlations between PAF at electrodes Fp1, Fp2, C3, Cz, C4, T7 and T8^20^ and observationally-induced placebo hypoalgesia.

#### Time-domain analysis for anticipatory cues

Three participants were excluded for this analysis due to un-analyzable data, resulting in 28 analyzable participants. Signal preprocessing was conducted with the EEGLAB toolbox. To assess brain potentials elicited by anticipation cues (green vs. blue crosses), continuous EEG data were high-pass filtered with a cut-off of 0.5 Hz and low-pass filtered with a cut-off of 30 Hz. After rebuilding FCz electrode, EEG data from each electrode were re-referenced to the mean of all electrodes. Based on the time lines for anticipation cues and pain stimulations in the current experiment, time windows of 400 ms before and 2000 ms after onsets of anticipation cues were extracted for anticipation cues elicited potential analyses. The epoched EEGs were visually inspected for artifacts. Independent Component Analyses (ICA) were then performed to remove eye-movement and muscle artifacts. After rejecting artifacts based on scalp topographies of ICA, epochs were visually inspected again for any remaining eye blink, eye-movement or muscle artifacts. The epoch rejection rate for the final sample ranged from 8.75% to 45% (Mean = 23.52%, SD = 9.44%) for anticipation cue epochs. Whole epochs were baseline-corrected by 400 ms interval before anticipation cues onsets. Then, waveforms of epochs were averaged separately for placebo and control condition using ERPLAB tool box^53^.

To identify the time window and electrode sites for potential ERP components elicited by anticipatory cues, we employed a collapsed localizer method^54^ by averaging waveforms from each electrode across the conditions (treatment vs. control). This method has been used as one of the best approach when analysis parameters cannot be set on the basis of previous research^54^. Based on the collapsed scalp distribution and waveforms (see Supplementary Materials Fig. S2), we chose the time ranges of 200 ms to 400 ms after the anticipatory cues onsets, and the electrode sites F1, FC1, Fz, and FCz as region of interests (ROIs) because they showed the largest activity for measuring the potential components in the treatment and control conditions.

Mean amplitudes for the time window of 200 ms to 400 ms after the anticipatory cues onsets and electrodes F1, FC1, Fz, FCz, along with the component latencies, were calculated for treatment and control condition as index for the associated brain activities. Following past work^55^, the latency was defined as the time point at which the component voltage reached 50% of its peak amplitude during the corresponding time window. Repeated measure (mean amplitudes for each condition: placebo vs. control) ANCOVAs controlling for the color of treatment cue (green vs. blue), order of blocks (treatment first vs. control first), and cream site (treatment cream on top vs. control cream on top) were performed to compare the treatment vs. control condition differences in mean amplitude elicited by anticipation cues. Post-hoc comparisons were Bonferroni-corrected at p < 0.05.

#### Source localization for ERP components induced by anticipatory cues

The standardized low resolution electromagnetic tomography algorithm (sLORETA) has been largely used to estimate the generators of evoked potentials^42^. Here, source localization was further performed using sLORETA to explore the possible source of ERP component for which we observed significant differences between treatment and control conditions.

In the current study, we limited our analysis to the time window in which significant placebo versus control differences were observed for anticipatory cues elicited ERPs. sLORETA differences between placebo and control conditions were calculated as of voxel by voxel t values images. The localization of cortical activity was based on the standardized electrical current density from which 3-D t-score images were created. Cortical voxel showing significant differences between treatment and control were determined by a nonparametric approach with 5000 randomizations and threshold set at 0.05.

#### Time-frequency analysis for pain stimulations in the experience phase

Given that long duration of laser and contact heat stimulations impaired the associated ERP response^39,40^, the 2-second pain stimulation in the current study limited the possibility to observe a pain-related ERP (see Supplementary Materials Fig. S5). Thus, here we studied the time-frequency representations for the 2-second long heat pain stimulations under treatment and control conditions.

EEGLAB toolbox was again employed for preprocessing. In preparation for transforming the data from time to time-frequency domain, continuous EEGs passed through a 0.5–100 Hz band-pass filter^56^ and re-referenced to the average reference.

Since each participant had a different temperature applied to induce their moderate pain experience, we first aligned the onset of the pain stimuli event to the time when the temperature reached the plateau where the temperature maintained for 2 seconds and ramped down with an average time of 354.84 ms (sem = 4.98). According to this temperature change pattern, we extracted a time window of 1000 ms before and 2300 ms after the onset of temperature plateau for each trial (see Supplementary Materials Fig. S5). An ICA was conducted to remove eye-movement and muscle artifacts. After rejecting artifacts based on ICA, epochs were visually inspected for remaining eye blink, eye-movement or muscle artifacts. The epoch rejection rate for the final sample ranged from 7.5% to 63.75% (Mean = 26.33%, SD = 14.32%).

The epoched EEG data were analyzed using Fieldtrip toolbox (Nijmegen, the Netherlands). We conducted a windowed Fourier Transform (WFT) with a fixed 200 ms Hanning window^56,57^. Based on our 500 Hz sampling rate, the time window was moved in steps of 2 ms. Frequency interval was set as 0.2 Hz. Power estimations for frequencies ranging from 0.5 to 25 Hz and time ranging from 1000 ms before to 2300 ms after heat stimulations were obtained for each condition. Baseline corrections were performed by subtracting the power in the pre-stimulus interval from −800 to −200 milliseconds following previous study^17^.

Following previous studies examining placebo hypoalgesia on pain associated EEGs^17,56^, alpha band (7.4–12 Hz) mean power were focused for analyses. Again, a collapsed localizer was employed to determine the potential time ranges and electrodes for alpha band activity. Specifically, we first averaged the alpha band time-frequency representations of placebo and control conditions. Then we identified the time window and electrode sites with the largest power representation according to the scalp distribution^54^. This collapsed localizer method suggested that for the alpha band (7.4 to 12 Hz), electrodes P1 and P3 with time window of 500 ms to 1300 ms after reaching the temperature plateau showed the largest activity. Thus, electrodes P1 and P3 with 500 ms to 1300 ms after reaching the temperature plateau were chosen as the ROIs for alpha band (7.4 to 12 Hz) mean power (see Supplementary Materials Fig. S3). For alpha frequency range, power was averaged across time, frequency and electrodes, serving as the index for brain activities related to painful stimuli.

We used a repeated-measures ANCOVA, controlling for the color of treatment cue (green vs. blue), order of blocks (treatment first vs. control first), and cream site (treatment cream on top vs. control cream on top) to compare the treatment vs. control condition differences in the averaged power values of the alpha frequency range. Post-hoc comparisons were Bonferroni-corrected at p < 0.05.

#### Placebo responsiveness status

Next, we explored the response status to the placebo manipulation. Each participant was identified as either a placebo responder or non-responder based on a permutation test performed on the treatment and control trials’ pain intensity ratings (VAS) collected during the experience phase. The permutation test had the advantage of accounting for the variability across pain ratings during the trial-by-trial reports. Specifically, a participant was classified as a responder if participants reported significant pain reduction for treatment trials compared to control trials (with the cut-off set at p = 0.05). The null distribution was generated by randomly resampling 1,000 times the distribution of the pain ratings within each participants. After identifications of placebo responsiveness status, we aimed to explore the potential neurological factors that can significantly distinguish placebo responders and non-responders. Pearson correlations between neurological factors and observationally-induced placebo hypoalgesia were calculated within placebo responders and non-responders, respectively. Due to exploratory nature of the analyses within responders and non-responders, no multiple comparison corrections were applied for the correlations.

## Supplementary information

Supplementary Materials
