# Supplementary material for: Neural and behavioral changes driven by observationally-induced hypoalgesia
Source: Sci Rep. 2019 Dec 24;9:19760. doi: 10.1038/s41598-019-56188-2 (PMC6930247; doi:10.1038/s41598-019-56188-2)
Supplement: Supplementary file 1 — Supplementary Materials [file 41598_2019_56188_MOESM1_ESM.docx]

**The Supplementary Table and Figures for**

**Title:** Neural and behavioral changes driven by observationally-induced hypoalgesia

Nandini Raghuraman^1§^, Yang Wang^1§^, Lieven A. Schenk^5^, Andrew J. Furman^3,4^, Christina Tricou^3^, David A. Seminowicz^3,4^, Luana Colloca^1, 2, 4*^

¹ Department of Pain and Translational Symptom Sciences University of Maryland School of Nursing,

² Department of Anesthesiology/Psychiatry University of Maryland School of Medicine,

^3^ Department of Neural Pain Sciences University of Maryland School of Dentistry,

^4^ UMB Center to Advance Chronic Pain Research**,**

^5^ Social Neuroscience Lab, Max Planck Society, Berlin, Germany

§ = Equal Contribution

**5 Figures**

Subject #___________

Date _________

Protocol # HP-00069094

Study Exit Form

When you were deciding whether to participate in this study, we explained to you that this study uses elements of deception because we were going to provide you with misleading information about parts of the study. When we use deception in a study, we always explain the nature and purpose of the deception after participation. The purpose of this form is to tell you the actual purpose of this study. We will then answer any questions you may have about this study.

You were told that the aims of this study are to understand the brain activity that occurs while observing someone else in pain. We told you that one of the creams we applied to your arm is a painkiller and that the other cream has no pain relieving effect. However, the truth is that neither one of the creams has pain relieving effect. The person you observed experiencing pain (i.e. the demonstrator) is a member of our research team. The demonstrator falsely rated their pain at certain numbers after the presentation of the blue and green color cues with the intention to mislead you to believe that one of the two creams had pain relieving effects, although it does not.

The reason we had to deceive you for this study was because we want to test if observing a demonstrator experiencing a certain intensity of pain would affect how you experienced pain. We were not able to tell you this information until you finished participating in this experiment as we needed you to think that one of the creams had potentially pain relieving effects.

Some people do not want to have any further involvement in a study once the deception of the study is described. Please answer the following question by selecting one of the answers below. Are we allowed to use your results as data for this study?

□ Yes, you may use my study results.

□ No, you may not use my study results.

If you are concerned or uncomfortable about the fact that you have been intentionally deceived, we would be happy to discuss this with you. Please contact the Principal Investigator (PI) Luana Colloca at 410-706-8422 or [colloca@umaryland.edu](mailto:colloca@umaryland.edu).

**Fig. S1. Pain rating reductions.** When compared to a no-intervention condition, pain reductions in the treatment trials were larger than the control trials. Differences were calculated between the no-intervention trial and the average for treatment and control trials of the testing phase. Data are expressed as delta scores (mean±sem).


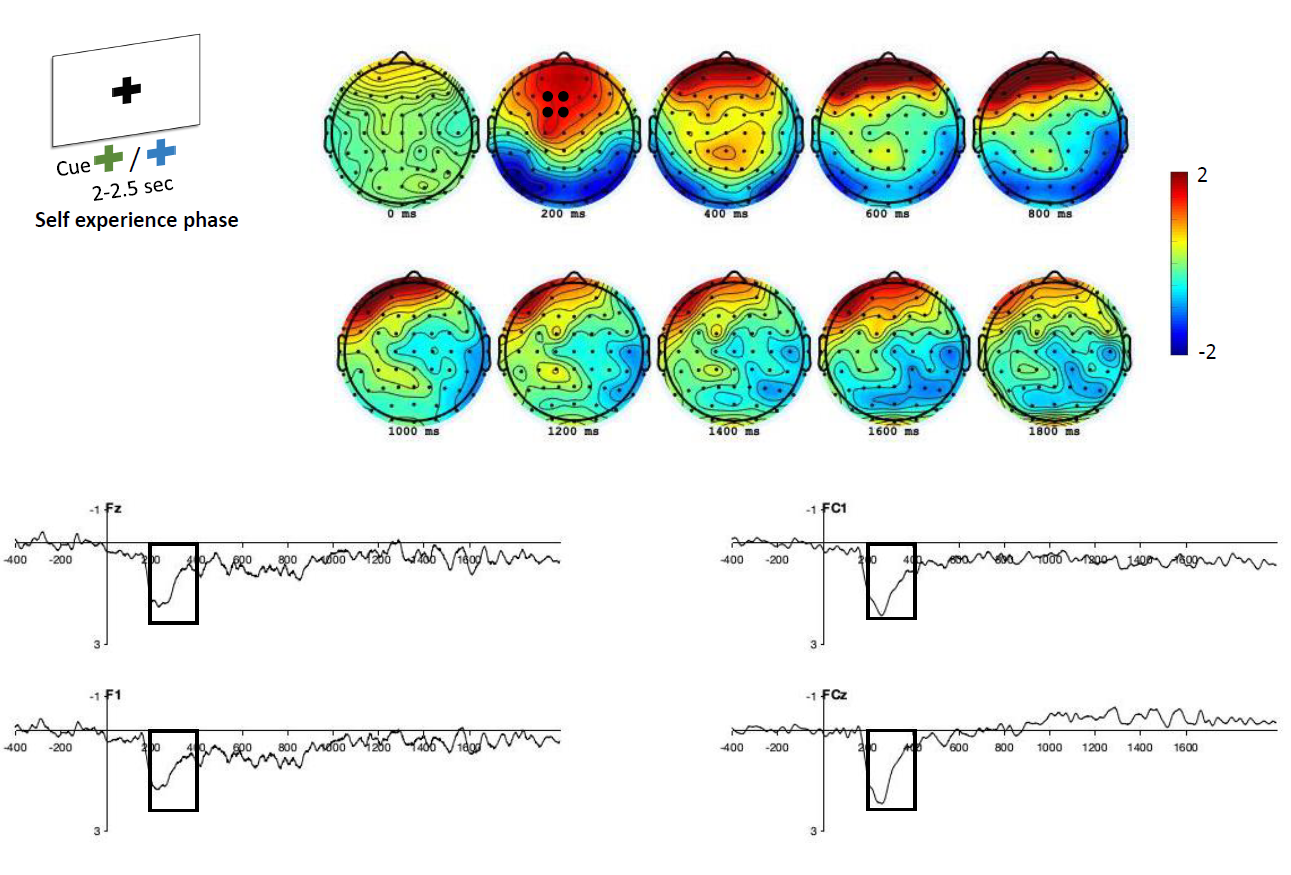


**Fig. S2. Collapsed Localizer identifying Region of Interests (ROIs) for anticipatory cue elicits potential.** To identify the time window and electrode sites for potential ERP components corresponding to anticipatory cues elicited potentials, we averaged waveforms from each electrode across the treatment and control conditions. Based on the collapsed scalp distribution and waveforms, we chose the time ranges and the electrode sites that showing the largest activity for measuring the ERP components. According to the collapsed localizer, electrodes F1, FC1, Fz and FCz with time window 200-400 ms after onset of the cues were identified for main analyses.


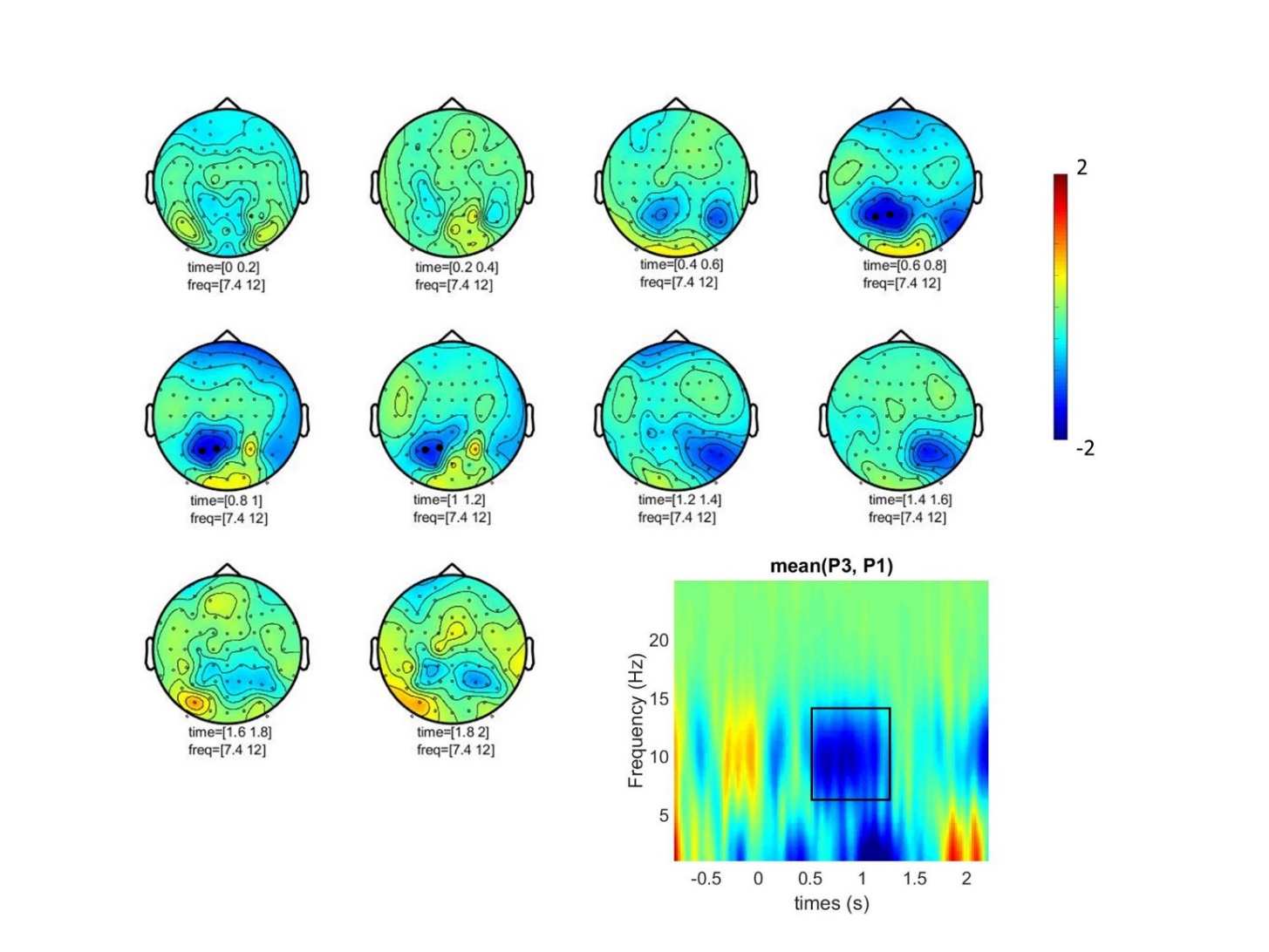


**Fig. S3. Collapsed localizer identifying ROIs for alpha band mean power during self-experienced pain.** To identify the time window and electrode sites for alpha band ROIs, time-frequency representations were averaged across the treatment and control conditions. Based on the averaged scalp distributions for each frequency range, time windows and electrode sites with the largest power representation were identified. Electrodes P1 and P3 with time window 500 to 1300 ms after reaching the peak temperature of heat pain stimuli were chosen for alpha band (7.4 to 12 Hz) mean power ROIs.

**Fig. S4.** **Pearson correlations between pain sensitivity and observationally-induced placebo hypoalgesia.** There were no significant correlations between observationally-induced hypoalgesia and temperature used for experiment, pain threshold or pain tolerance, suggesting that the observationally-induced hypoalgesia was independent of individual differences in pain sensitivity.

**Frequency (0.5 to 25 Hz)**

0.5 to 1.3 s

**Control**


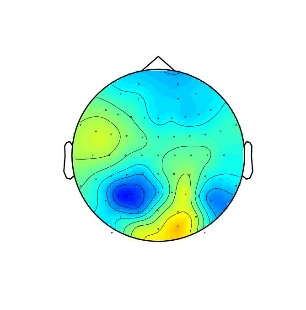

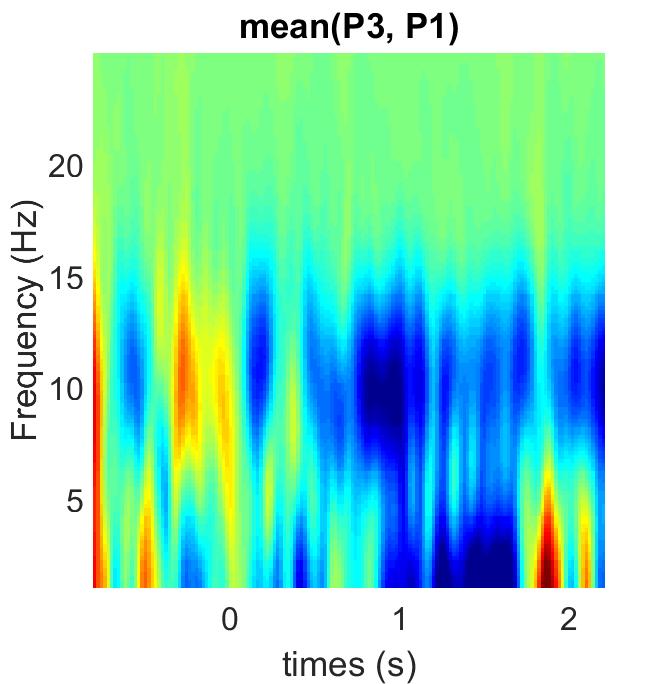


**Treatment**


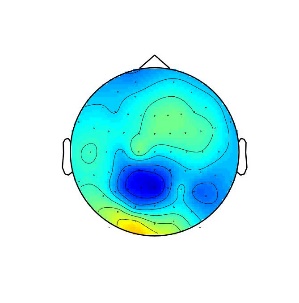


0.5 to 1.3 s


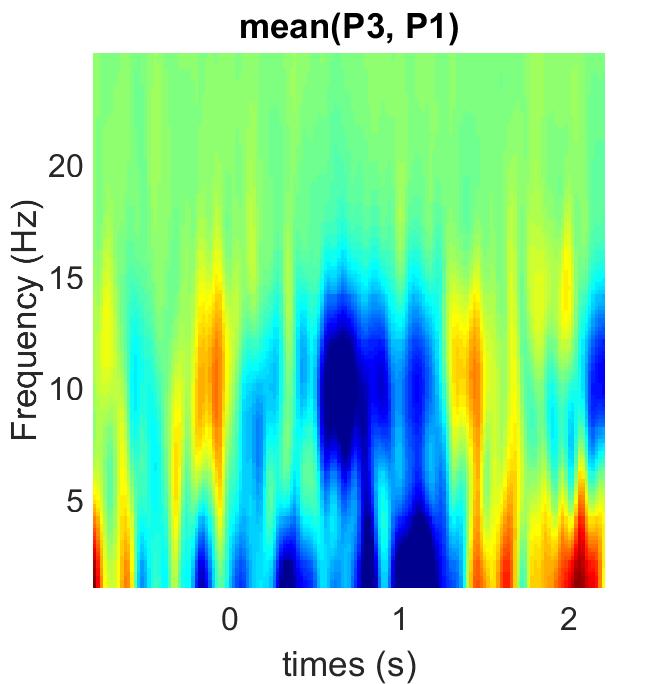


times (s)

mean (P1, P3)

**Frequency (0.5 to 25 Hz)**

times (s)

mean (P1, P3)

a

b


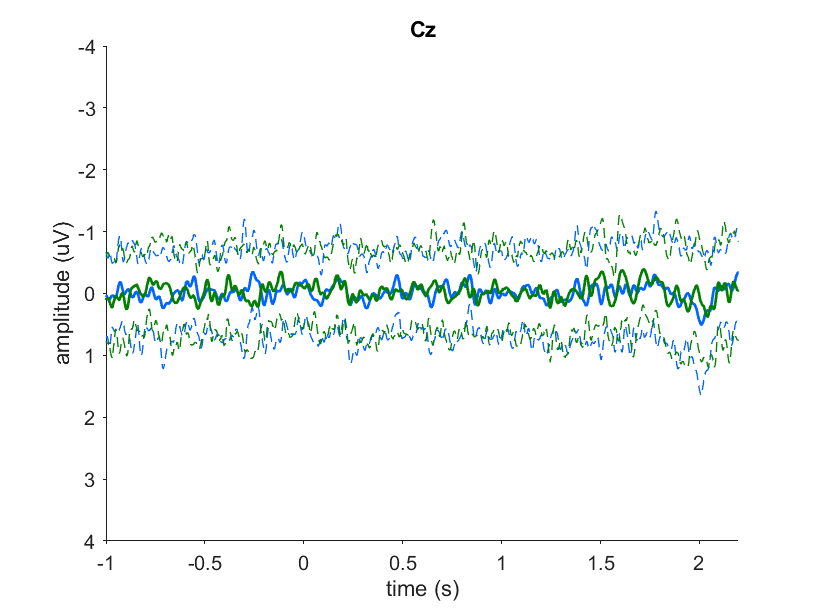


**Treatment cue**

**Control cue**

**SD**

**Temperature**

**Fig. S5.** **Heat pain stimulations related time-frequency representation and ERP response.** **(a)** Alpha suppression was observed at occipital areas of the brain (P1 and P3) in both control and treatment conditions at latency about 500 to 1300 ms There was no differences on alpha band mean power between treatment and control condition.  **(b)** The golden line was the temperature changes inducing heat pain experiences. Each participant was applied to the temperature that induced moderate pain experience (i.e., self-report pain of 50-60 based on the 100 VAS scale). The temperature ramp up rate was 70 Celsius degree/s and ramp down rate was 40 Celsius degree/s. In the current study, the mean ramp up time duration was 202.77 ms (sem=2.84), and the mean ramp down time was 354.84 ms (sem=4.98). The temperature maintained plateau for 2 seconds. According to this temperature changes pattern, we first align the onset of the pain stimuli event to the time when the temperature reached the plateau, then extracted a time window of 1000 ms before and 2300 ms after the onset of temperature plateau for each pain trial. Electrode Cz was chosen for pain elicited ERP analysis following previous studies^1,2^. We did not observe any heat pain induced ERP in the current study.

1 Wager, T. D., Matre, D. & Casey, K. L. Placebo effects in laser-evoked pain potentials. *Brain Behav Immun* **20**, 219-230, doi:10.1016/j.bbi.2006.01.007 (2006).

2 Hu, L., Mouraux, A., Hu, Y. & Iannetti, G. D. A novel approach for enhancing the signal-to-noise ratio and detecting automatically event-related potentials (ERPs) in single trials. *Neuroimage* **50**, 99-111, doi:10.1016/j.neuroimage.2009.12.010 (2010).
